# Retrospective study of postoperative pleural effusion with hypoxemia in critically ill pancreatic surgery patients: model development and restricted cubic spline analysis

**DOI:** 10.7717/peerj.20635

**Published:** 2026-01-30

**Authors:** Bin Wang, Jie Zhao, Shuguang Yang, Xiaojiang Liu, Fengxue Zhu

**Affiliations:** 1 Department of Critical Care Medicine, Peking University People’s Hospital, Beijing, China; 2 Trauma Medicine Center, Peking University People’s Hospital, Beijing, China

**Keywords:** Pancreatic surgery, Pleural effusion, Predictive model, Restricted cubic spline analysis

## Abstract

**Background:**

Pleural effusion is a common postoperative complication following pancreatic surgery. It is associated with hypoxemia, often requiring prolonged mechanical ventilation and contributing to adverse clinical outcomes. Identifying risk factors and developing predictive models in critically ill patients after pancreatic surgery may facilitate early recognition and guide timely interventions to improve prognosis.

**Methods:**

We retrospectively reviewed 518 intensive care unit (ICU)-admitted patients who underwent pancreatic surgery at Peking University People’s Hospital from January 2016 to June 2024. Patients were grouped by postoperative pleural effusion status. Least absolute shrinkage and selection operator (LASSO)-logistic was used to identify key predictors and guide model development. Internal validation was conducted using 1,000 bootstrap resamples. Model discrimination and calibration were assessed using receiver operating characteristic (ROC) curve (area under the curve, AUC) and calibration plots. Decision curve analysis evaluated clinical utility, while restricted cubic spline analysis was applied to explore nonlinear effects of continuous predictors.

**Results:**

Among 518 patients, 144 developed postoperative pleural effusion. Independent predictors included age, body mass index (BMI), atrial fibrillation, American Society of Anesthesiologists (ASA) grade, and intraoperative transfusion. A nomogram-based model incorporating these variables demonstrated good discrimination (AUC = 0.733, 95% CI [0.683–0.783]) and reliable calibration. Decision curve analysis confirmed clinical utility across a range of threshold probabilities. Restricted cubic spline analysis revealed nonlinear associations: age-related risk rose sharply beyond 65 years, while BMI showed a U-shaped relationship, with elevated risk below and above the inflection point of 22.6.

**Conclusion:**

This study developed a predictive model for postoperative pleural effusion in critically ill patients undergoing pancreatic surgery using LASSO-logistic regression. The model demonstrated robust discrimination and calibration, highlighting its potential utility in early risk stratification and individualized clinical decision-making.

## Introduction

Pancreatic diseases, including acute and chronic pancreatitis as well as pancreatic tumors, are prevalent clinical conditions (Sevim, Chela & Ertugrul, 2023). Pancreatic surgery is characterized by its technical complexity and high degree of difficulty. Postoperative complications occur in 30% to 50% of cases, underscoring the importance of meticulous perioperative management and surgical expertise (Alhulaili et al., 2025). Common complications following pancreatic surgery include pancreatic leakage, hemorrhage, infection, diabetes, and indigestion are commonly observed following pancreatic surgery. These adverse events not only exacerbate patient discomfort, but also hinder recovery and negatively impact long-term survival outcomes.

Pleural effusion is another common postoperative complication following pancreatic surgery. It can contribute to hypoxemia, often necessitating prolonged mechanical ventilation and adversely affecting patient prognosis (Wang et al., 2025a). Pleural effusion frequently arises following pancreatic surgery due to the anatomical location of the pancreas, the complexity of surgical procedures and significant pathophysiological disturbances. The pancreas is a retroperitoneal organ situated adjacent to the diaphragm, a key anatomical structure that separates the thoracic and abdominal cavities. Inflammatory processes or exudative fluid originating from the pancreas can readily spread into the thoracic cavity *via* potential weak points in the diaphragm, such as the esophageal and aortic hiatuses. The pancreatic lymphatic vessels communicate with the thoracic lymphatic system through interconnected branches. Following pancreatic surgery, inflammatory responses or leakage of pancreatic juice may disseminate into the thoracic cavity *via* these lymphatic pathways (Ponikowska et al., 2025). Pancreatic surgery is inherently complex and typically involves prolonged operative time. The procedure requires substantial manipulation such as traction and compression of abdominal organs, which can readily induce postoperative inflammatory responses. The pancreas contains abundant digestive enzymes, and surgical injury to pancreatic tissue may result in the leakage of pancreatic juice into the abdominal cavity. Owing to its highly corrosive nature, pancreatic juice can damage abdominal and thoracic tissues, triggering inflammatory responses and promoting the formation of exudative fluid (Khadka et al., 2024).

While minor pleural effusion may have limited clinical impact, its occurrence, in conjunction with hypoxemia, can significantly increase intensive care unit (ICU) stays, hospitalization costs, and mortality (Bediwy et al., 2023). This study aims to investigate the occurrence of pleural effusion accompanied by hypoxemia in critically ill patients following pancreatic surgery. It further analyzes the associated risk factors and develops a predictive model to facilitate early identification and timely clinical intervention.

## Materials and Methods

### Research subjects

**Figure 1:**
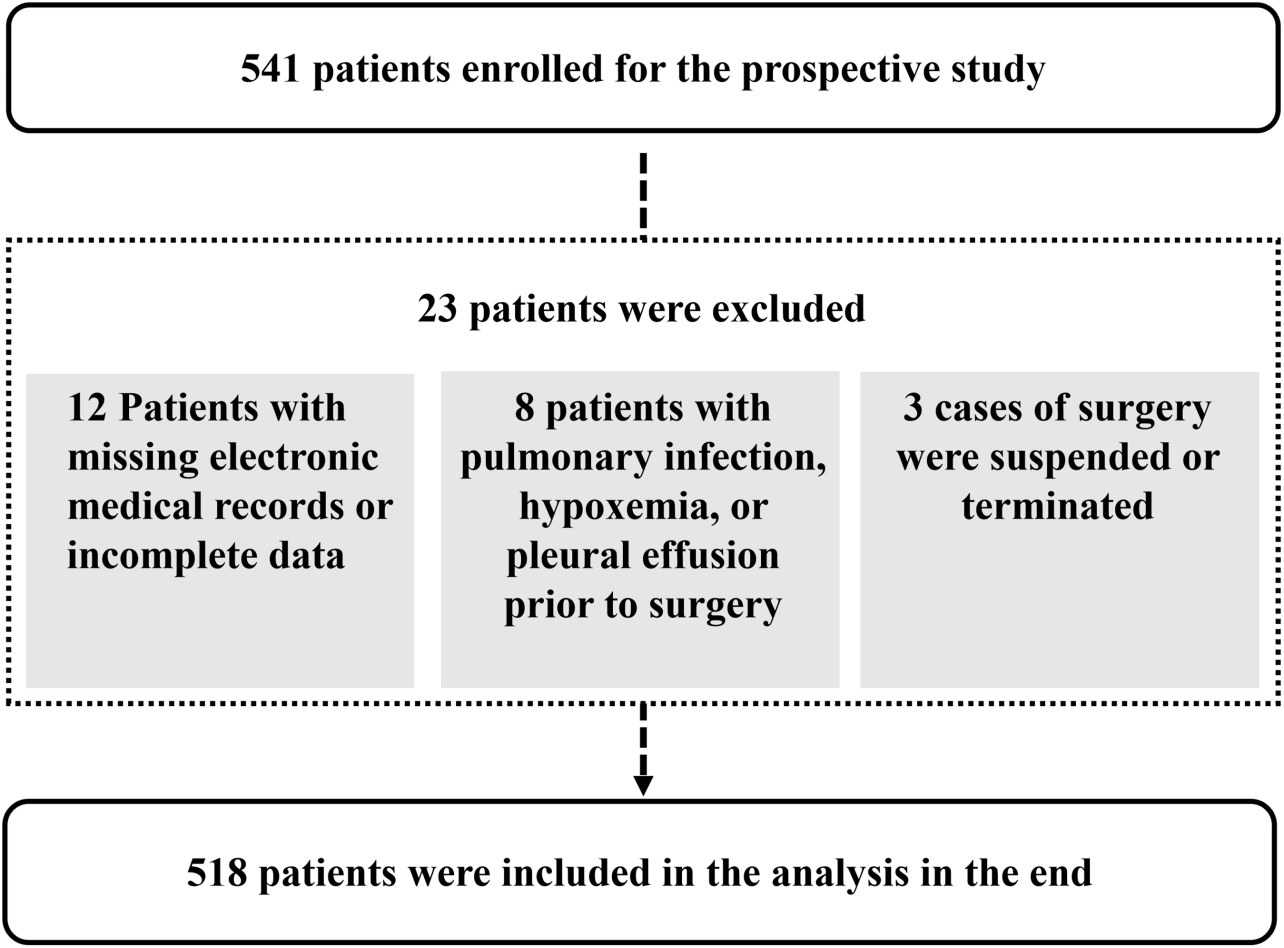
Study flowchart.

This is a retrospective cohort study. A total of 518 patients who underwent pancreatic surgery and were admitted to the Department of Critical Care Medicine at Peking University People’s Hospital between January 2016 and June 2024 were included in this study. Inclusion criteria: (1) Age ≥18 years, (2) direct admission to the ICU following pancreatic surgery, and (3) Acute Physiology and Chronic Health Evaluation II (APACHE II) score ≥10. Exclusion criteria: (1) Patients with missing electronic medical records or incomplete data, (2) evidence of pulmonary infection, hypoxemia, or pleural effusion prior to surgery, (3) cases where surgery was suspended or terminated due to acute events such as respiratory or cardiac arrest (Fig. 1).

Inclusion criteria were designed to ensure a clinically relevant and homogeneous study population. Specifically, patients aged ≥18 years who were directly admitted to the ICU following pancreatic surgery and had an APACHE II score ≥10 were included. These criteria aimed to capture adult patients with significant physiological stress, thereby enhancing the applicability of the predictive model to critically ill surgical populations.

Exclusion criteria were applied to minimize confounding and ensure the accuracy of postoperative pleural effusion attribution. Patients with incomplete electronic medical records were excluded to maintain data integrity. Those with preoperative evidence of pulmonary infection, hypoxemia, or pleural effusion were excluded to avoid misclassification of pre-existing conditions as postoperative complications. Additionally, cases in which surgery was suspended or terminated due to acute intraoperative events (*e.g*., cardiac or respiratory arrest) were excluded, as these patients represent a distinct clinical trajectory not suitable for predictive modeling of standard postoperative outcomes.

These inclusion and exclusion criteria were carefully selected to balance clinical relevance and data quality, thereby supporting the robustness of the model development process.

This study adhered to the ethical principles set forth in the Declaration of Helsinki and was approved by the Ethics Committee of Peking University People’s Hospital (Approval No. 2023PHB071-001). Given its retrospective design, the requirement for informed consent was waived.

### Study variables

A comprehensive dataset was compiled to evaluate prognostic factors in surgical patients. Demographic data included gender, age, and body mass index (BMI). Medical history variables encompassed chronic obstructive pulmonary disease (COPD), cardiac insufficiency, atrial fibrillation, diabetes mellitus (includes both type 1 and type 2 diabetes), hypertension, coronary heart disease, cerebral infarction, chemotherapy, hepatitis B virus (HBV) infection, fatty liver disease, and smoking status. Preoperative laboratory assessments included leukocyte count, hemoglobin concentration, platelet count, serum albumin, total bilirubin, and creatinine levels. Postoperative measurements comprised creatine kinase-MB (CKMB), B-type natriuretic peptide (BNP), and lactic acid. Surgical and intraoperative factors recorded were type of surgery (*e.g*., laparotomy), multisite surgical involvement, intraperitoneal chemotherapy, American Society of Anesthesiologists (ASA) grade, duration of surgery, total intraoperative fluid intake and output, blood loss, urine volume, transfusion requirement, analgesic pump usage, and intraoperative norepinephrine administration. Key prognostic indicators included in-hospital mortality, ICU length of stay, total hospitalization duration, and overall cost.

In our study, pleural effusion was defined as the development of postoperative pleural fluid accumulation accompanied by hypoxemia. Radiographic diagnosis was based on blunting of the costophrenic angle, indistinct visualization of the ipsilateral costophrenic contour, and displacement of adjacent anatomical structures observed on upright chest radiographs (Miskovic & Lumb, 2017). Hypoxemia was defined as partial pressure of oxygen (PaO_2_) less than 60 mmHg on room air and/or a partial pressure of carbon dioxide greater than 50 mmHg, or a modified oxygenation index (PaO_2_/FiO_2_) less than 300 mmHg (Miskovic & Lumb, 2017).

To ensure clinical relevance and diagnostic consistency, the outcome of this study was defined as postoperative pleural effusion accompanied by hypoxemia. While minor or reactive pleural effusions are relatively common after abdominal surgery and often resolve spontaneously without clinical intervention, they typically do not warrant intensive care or targeted management. In contrast, pleural effusion with concurrent hypoxemia reflects a more severe physiological disturbance that may prolong mechanical ventilation, increase ICU burden, and adversely affect prognosis. Therefore, the study specifically focused on this symptomatic subset to better capture clinically meaningful complications and guide early risk stratification.

### Statistical method

Statistical analyses were conducted using R software (version 4.4.2; R Core Team, 2024). Continuous variables were presented as mean ± standard deviation (SD) or median with interquartile range (IQR), depending on their distributional characteristics. Categorical variables were summarized as frequencies and percentages. To reduce dimensionality and identify potential predictors, the least absolute shrinkage and selection operator (LASSO) regression was applied. Subsequently, multivariate logistic regression was performed to determine independent risk factors for postoperative pleural infection. A nomogram was constructed to visualize the predictive model, and its discriminatory performance was evaluated using the area under the receiver operating characteristic (ROC) curve. Model calibration and clinical applicability were further assessed *via* calibration plots and decision curve analysis (DCA). In addition, dose-response relationships between continuous predictors and the risk of pleural effusion were explored using restricted cubic spline (RCS) modeling. Statistical significance was defined as a *p*-value < 0.05.

## Results

A total of 518 patients were included in the study, comprising 144 individuals in the pleural effusion group and 374 in the non-effusion group. The overall incidence of postoperative pleural effusion was 27.8%. Patients in the pleural effusion group demonstrated a significantly higher in-hospital mortality rate, longer ICU stays, extended overall hospitalization duration, and greater hospitalization costs compared to those without pleural effusion (Table 1).

**Table 1:** Comparison of prognostic data in patients.

Prognostic indicator	Pleural effusion group ( *n* = 144)	Non-pleural effusion group ( *n* = 374)	Statistical value	*p*
In-hospital mortality (*n* (%))	13 (9.0)	11 (2.9)	χ^2^ = 8.717	0.003*
Length of stay in ICU (days, M (QL, QU))	3 (2, 5)	2 (2, 2)	Z = −9.247	<0.001*
Total length of stay (days, M (QL, QU))	31 (21, 46)	23 (17, 31)	Z = −5.401	<0.001*
Total cost (thousand yuan, M (QL, QU))	273,841 (187,316, 403,720)	170,714 (138,234, 233,489)	Z = −8.687	<0.001*

**Note:**

* *p* < 0.05; ICU: intensive care unit.

### Univariate analysis

Univariate analysis revealed that several factors were statistically associated with postoperative outcomes (*p* < 0.1). These included age, body mass index (BMI), atrial fibrillation, hypertension, cerebral infarction, preoperative leukocyte count, platelet count, total bilirubin, and postoperative lactic acid levels. Additionally, intraoperative characteristics such as ASA grade, operation duration, total fluid intake and output, blood loss, urine volume, and transfusion requirement showed significant differences (Table 2).

**Table 2:** Comparison of data between the two groups.

Factors	Pleural effusion group (*n* = 144)	Non-pleural effusion group (*n* = 374)	Statistical value	*p*
Gender (male/female)	92/52	224/150	χ^2^ = 0.698	0.404
Age (years, $\bar x$ ± *s*)	67.21 ± 10.46	62.93 ± 10.22	t = 4.087	<0.001*
BMI (kg/m^2^, $\bar x$ ± *s*)	23.51 ± 4.14	22.52 ± 3.12	t = 2.637	0.010*
**Comorbidities and past medical history**				
COPD (*n* (%))	18 (12.5)	35 (9.35)	χ^2^ = 1.117	0.291
Cardiac insufficiency (*n* (%))	2 (1.4)	4 (1.1)	χ^2^ = 0.093	0.761
Atrial fibrillation (*n* (%))	14 (9.7)	5 (1.3)	χ^2^ = 20.689	<0.001*
Diabetes mellitus (*n* (%))	43 (29.9)	90 (24.1)	χ^2^ = 1.831	0.176
Hypertension (*n* (%))	66 (45.8)	135 (36.1)	χ^2^ = 4.151	0.042*
Coronary heart disease (*n* (%))	9 (6.3)	23 (6.1)	χ^2^ = 0.002	0.966
Cerebral infarction (*n* (%))	15 (10.4)	15 (4.0)	χ^2^ = 7.820	0.005*
Chemotherapy (*n* (%))	2 (1.3)	3 (0.8)	χ^2^ = 0.374	0.541
HBV infection (*n* (%))	4 (2.6)	15 (4.0)	χ^2^ = 0.447	0.504
Fatty liver disease (*n* (%))	4 (2.6)	20 (5.3)	χ^2^ = 1.554	0.213
Smoke (*n* (%))	43 (29.9)	126 (33.7)	χ^2^ = 0.693	0.405
**laboratory examination**				
Preoperative leukocyte (×10^9^/L, M (Q_1_, Q_3_))	6.1 (5.0, 8.4)	5.7 (4.7, 7.2)	Z = −1.791	0.073*
Preoperative hemoglobin (g/L, M (Q_1_, Q_3_))	121 (105, 133)	125 (112, 135)	Z = −1.490	0.136
Preoperative platelet (×10^9^/L, M (Q_1_, Q_3_))	223 (174, 281)	204 (159, 261)	Z = −1.737	0.082*
Preoperative albumin (×10^9^/L, M (Q_1_, Q_3_))	37.4 (34.1, 40.4)	37.8 (35, 40.5)	Z = −1.261	0.207
Preoperative total bilirubin (umol/L, M (Q1, Q3))	81.7 (15.5, 206.1)	32.3 (12.2, 154.9)	Z = −2.557	0.011*
Preoperative creatinine (umol/L, M (Q1, Q3))	62.5 (53.8, 77)	64 (54, 77)	Z = −0.244	0.807
Postoperative CK-MB (ng/ml, M (Q1, Q3))	1.7 (1.1, 2.6)	1.5 (1.0, 2.5)	Z = −1.433	0.152
Postoperative BNP (pg/ml, M (Q1, Q3))	85.5 (45.8, 155.5)	84 (45, 145.3)	Z = −0.874	0.382
Postoperative lactic acid (mmol/L, M (Q1, Q3))	1.2 (0.9, 1.8)	1 (0.8, 1.6)	= −2.961	0.003*
**Surgery and anesthesia**				
Laparotomy surgery (*n* (%))	46 (31.9)	115 (30.7)	χ^2^ = 0.057	0.812
Multisite surgery (*n* (%))	134 (93.1)	242 (64.7)	χ^2^ = 0.362	0.547
Intraperitoneal chemotherapy (*n* (%))	27 (18.8)	64 (17.1)	χ^2^ = 0.193	0.661
ASA grade (level, M (Q_1_, Q_3_))	2 (2, 3)	2 (2, 2)	Z = −4.646	<0.001*
Operation duration (min, M (Q_1_, Q_3_))	424 (360, 501)	389 (331, 479)	Z = −2.729	0.006*
Total intraoperative intake (ml, M (Q1, Q3))	5,470 (4,390, 6,803)	4,800 (3,900, 5,878)	Z = −3.994	<0.001*
Total intraoperative output (ml, M (Q1, Q3))	2,075 (1,400, 2,850)	1,778 (1,200, 2,400)	Z = −3.119	0.002*
Intraoperative blood loss (ml, M (Q_1_, Q_3_))	800 (500, 1,200)	650 (400, 1,000)	Z = −2.610	0.009*
Intraoperative urine volume (ml, M(Q_1_, Q_3_))	1,100 (800, 1,600)	1,000 (700, 1,363)	Z = −1.809	0.070*
Intraoperative transfusion (*n* (%))	85 (59.0)	142 (38.0)	χ^2^ = 18.731	<0.001*
Analgesic pump (*n* (%))	49 (34.0)	129 (34.5)	χ^2^ = 0.010	0.921
Intraoperative norepinephrine infusion (*n* (%))	13 (9.0)	25 (6.7)	χ^2^ = 0.840	0.359

**Note:**

* *p* < 0.05; BMI, body mass index; COPD, chronic obstructive pulmonary disease; HBV, Hepatitis B Virus; CK-MB, creatine kinase MB; BNP, B type natriuretic peptide; ASA, American Society of Anesthesiologists.

### LASSO regression analysis

**Figure 2:**
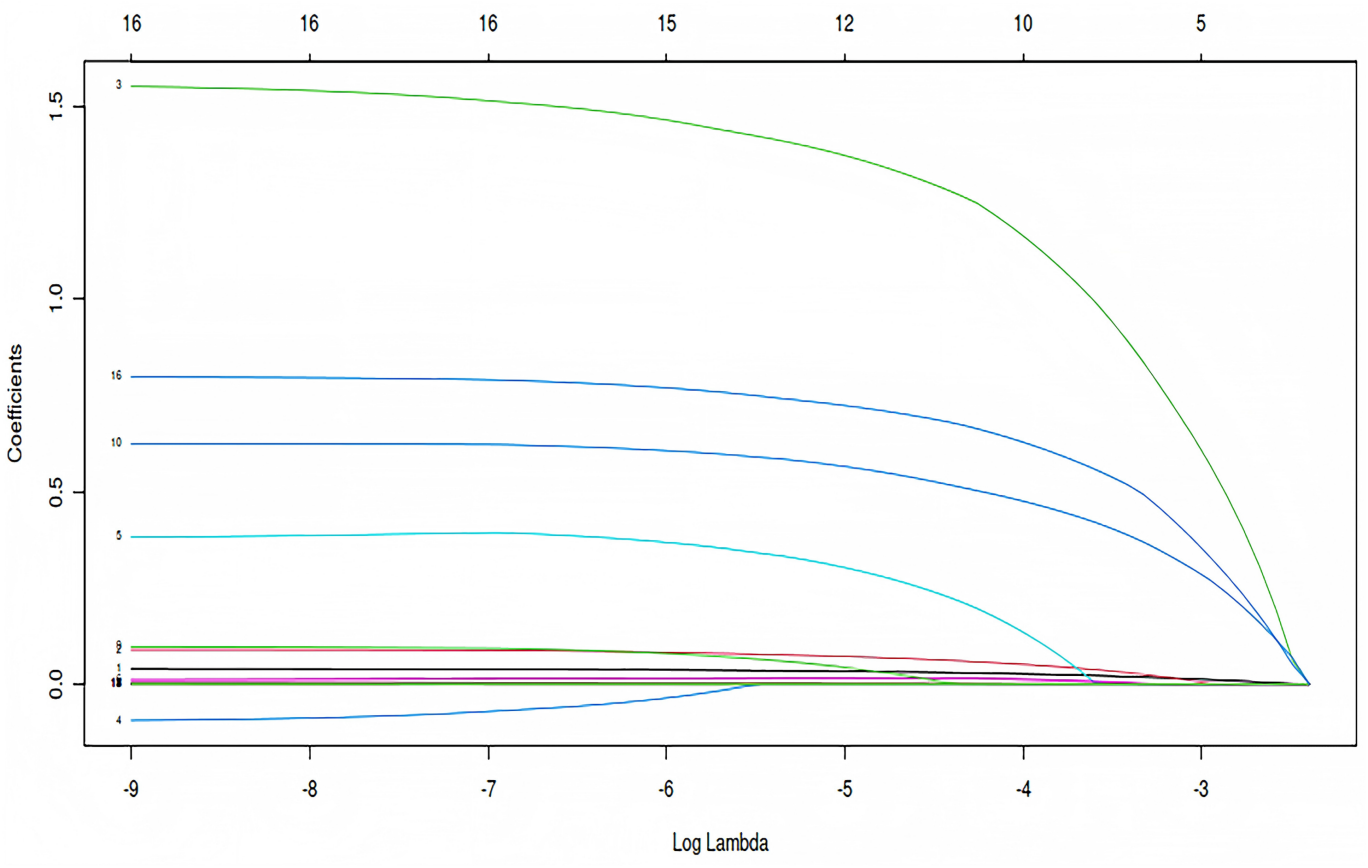
Coefficient trajectories across log-transformed λ values in LASSO regression. How regression coefficients change as the regularization parameter λ increases in LASSO regression. The x-axis represents log(λ), and the y-axis shows the magnitude of each coefficient. Each colored line corresponds to a specific predictor variable.

**Figure 3:**
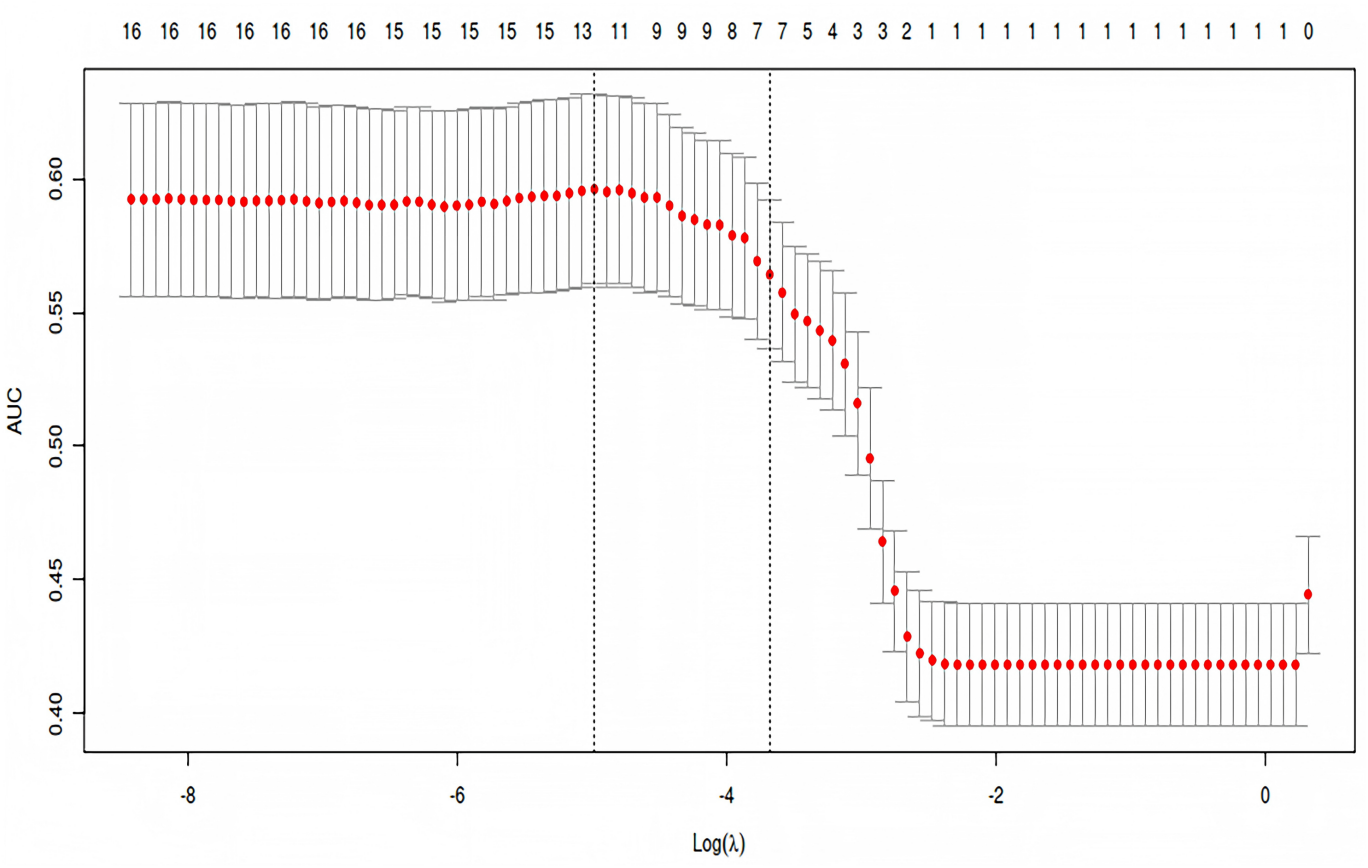
Cross-validation performance curve for selecting optimal λ in LASSO regression. Mean AUC values are plotted against log(λ), with error bars showing variability. Dashed lines indicate λ values for optimal performance and model simplicity. Numbers above the curve represent the count of non-zero coefficients at each λ.

LASSO regression analysis was employed to further reduce dimensionality and select relevant variables for pleural effusion prediction. Using a penalization parameter of λ = 0.0252274158479212, the model identified indices with non-zero coefficients as potentially informative (Fig. 2). This λ value was determined through cross-validation to balance model complexity and predictive accuracy, ensuring that less informative variables were penalized toward zero while retaining key predictors. The regularization process enhances model interpretability and reduces the risk of overfitting, thereby contributing to a more robust analysis. A total of ten variables were selected for subsequent multivariate analysis: age, BMI, atrial fibrillation, cerebral infarction, preoperative leukocyte count, platelet count, albumin level, ASA grade, total intraoperative fluid intake, and intraoperative blood loss (Fig. 3).

### Multivariate logistic regression analysis

Multivariate logistic regression analysis identified five independent risk factors significantly associated with postoperative pleural effusion: advanced age, higher BMI, atrial fibrillation, higher ASA grade, and intraoperative blood transfusion (Table 3).

**Table 3:** Multivariate logistic regression analysis.

Variable	B	S.E.	Z	OR	OR (95% CI)	*p*
Age	0.039	0.012	9.862	1.040	[1.015–1.066]	0.002*
BMI	0.087	0.033	6.826	1.091	[1.022–1.164]	0.009*
Atrial fibrillation	1.605	0.562	8.145	4.978	[1.653–14.987]	0.004*
Cerebral infarction	0.440	0.436	1.018	1.552	[0.661–3.646]	0.313
Preoperative leukocyte	0.023	0.040	0.329	1.023	[0.947–1.105]	0.567
Preoperative platelet	0.002	0.001	1.889	1.002	[0.999–1.005]	0.169
Preoperative total bilirubin	0.002	0.001	3.369	1.002	[1.000–1.003]	0.066
ASA grade	0.587	0.217	7.345	1.799	[1.176–2.751]	0.007*
Total intraoperative intake	0.000	0.000	2.014	1.000	[1.000–1.000]	0.156
Intraoperative transfusion	0.777	0.245	10.036	2.174	[1.345–3.516]	0.002*

**Note:**

* *p* < 0.05; BMI, body mass index; ASA, American Society of Anesthesiologists.

### Establishment of the predictive model

**Figure 4:**
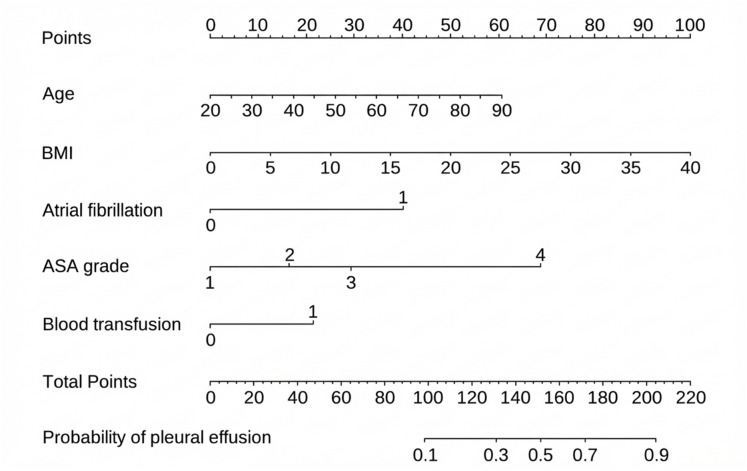
Nomogram for predicting pleural effusion based on selected predictors. Visual tool for estimating the probability of pleural effusion using multiple predictors. Each variable is assigned a point value according to its contribution in the model. The total score corresponds to a predicted probability on the bottom scale. To use the nomogram, locate the patient’s value for each predictor, read the corresponding points, sum them, and map the total to the outcome probability. BMI: body mass index.

**Figure 5:**
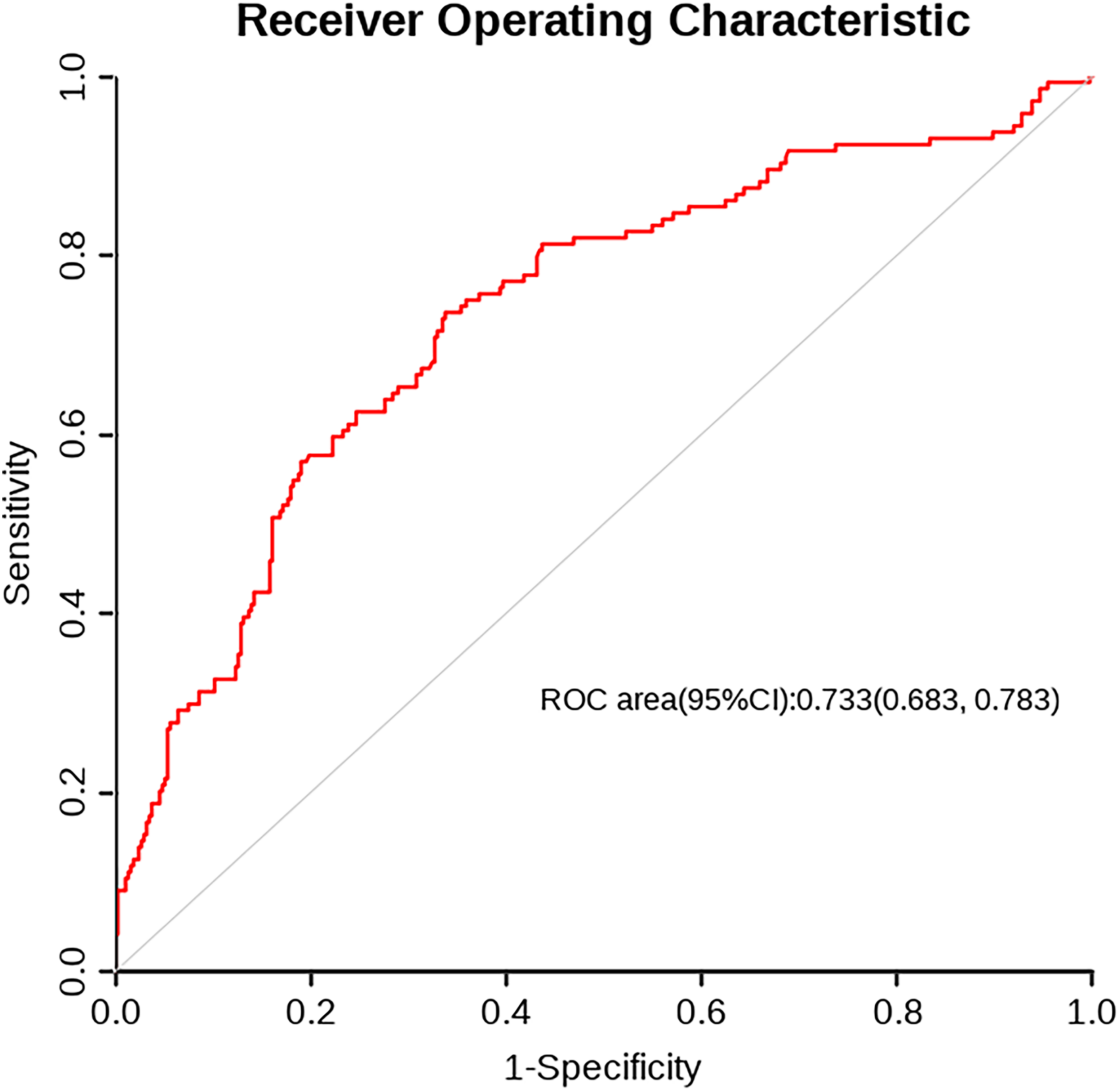
ROC curve. The trade-off between sensitivity and 1-specificity for the classification model. The area under the curve (AUC) is 0.733 (95% CI [0.683–0.783]), indicating fair discrimination. The curve above the diagonal suggests predictive value beyond random chance.

**Figure 6:**
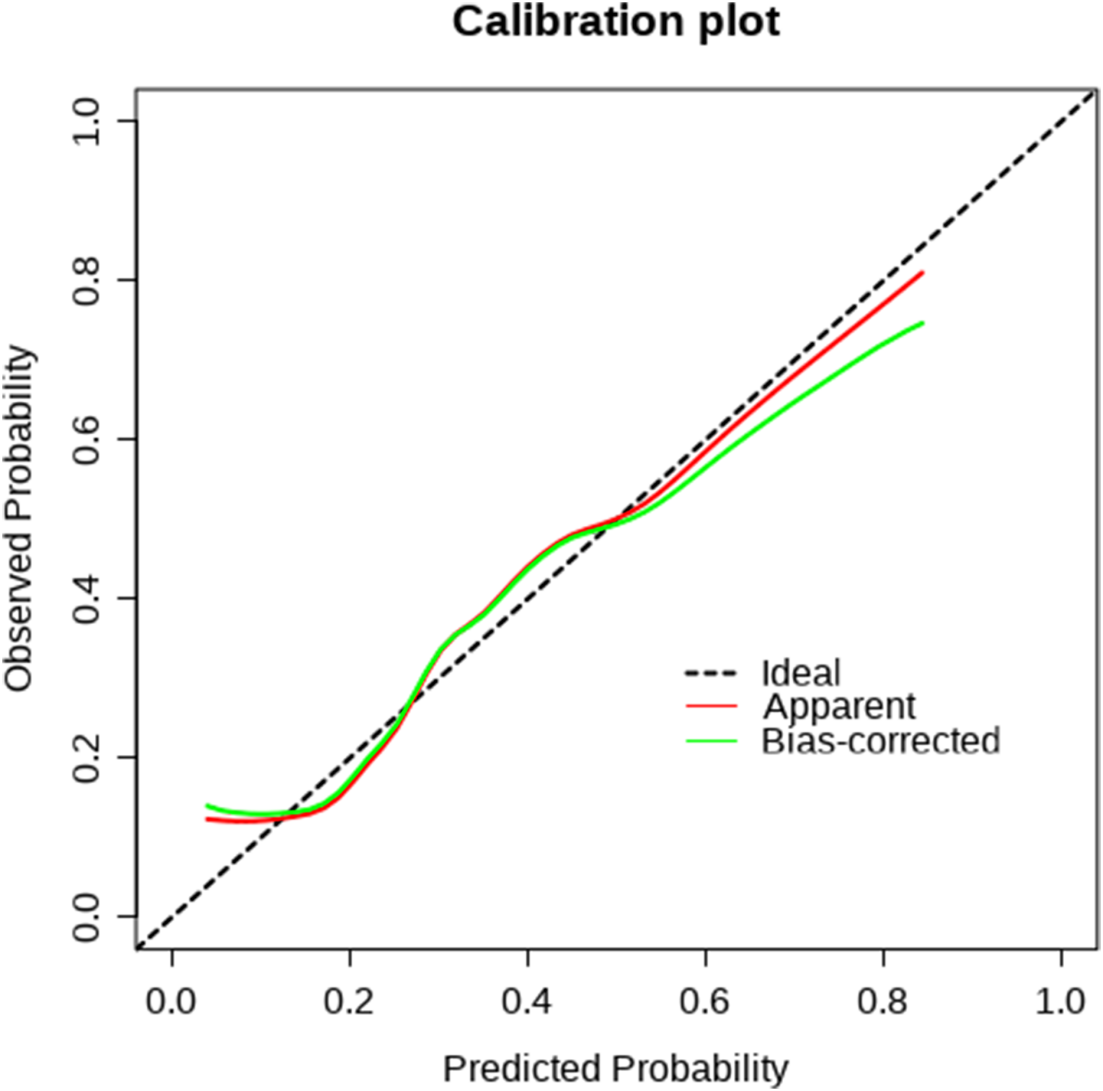
Calibration curve. The agreement between predicted probabilities and observed outcomes. A curve close to the diagonal line indicates good calibration and reliable prediction accuracy.

**Figure 7:**
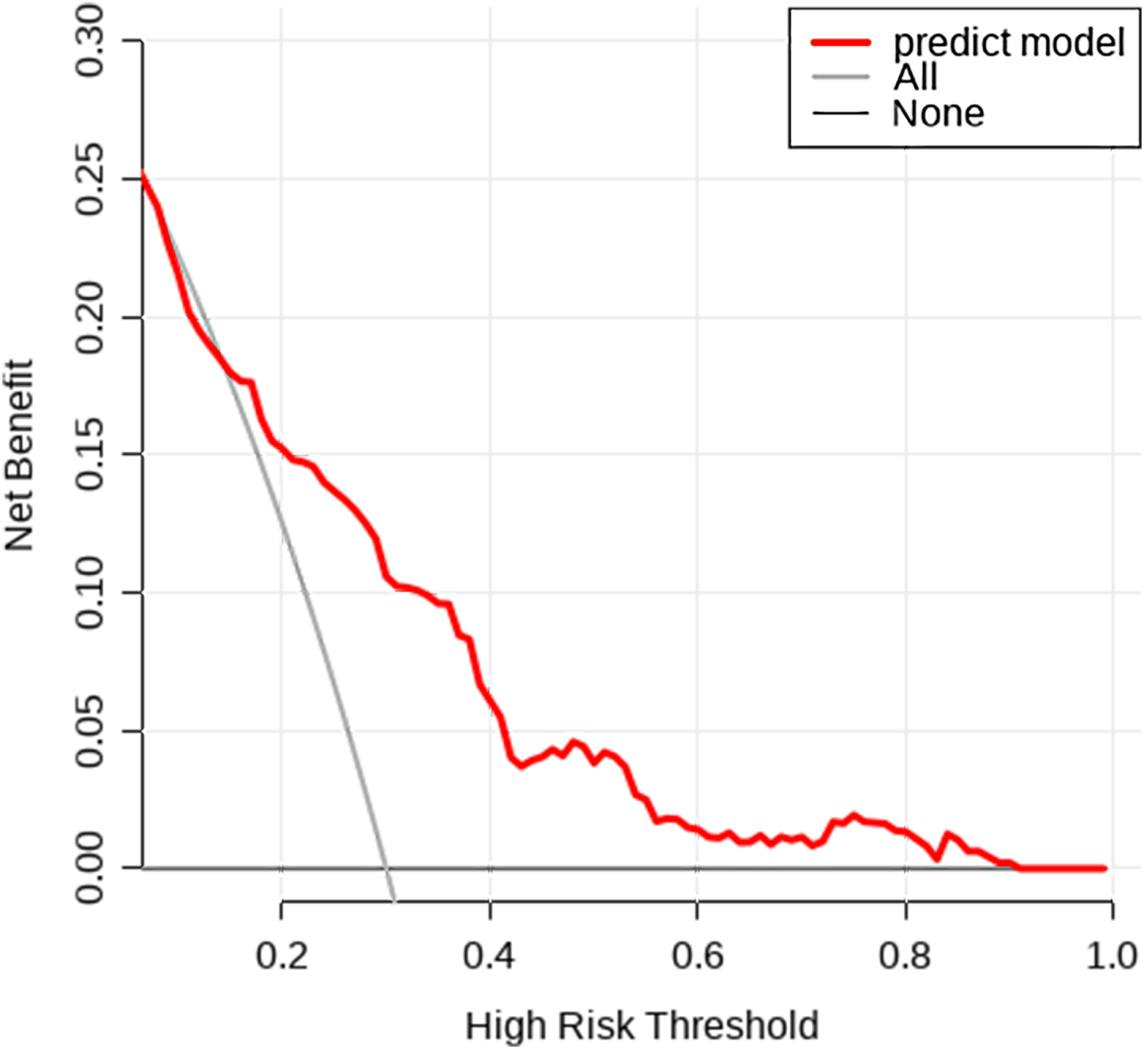
Decision curve analysis. The net clinical benefit of the prediction model across a range of threshold probabilities. Compared to the “All” and “None” strategies, the model shows greater net benefit within the clinically relevant threshold range.

A nomogram was developed based on five independent risk factors to predict the occurrence of postoperative pleural effusion (Fig. 4). The model demonstrated good discriminatory ability, with an area under the ROC curve (AUC) of 0.733 (95% CI [0.683–0.783]) (Fig. 5). Calibration plots revealed strong concordance between predicted and observed probabilities (Fig. 6). Furthermore, DCA confirmed the model’s clinical utility, showing superior net benefit compared to the “Treat All” and “Treat None” strategies (Fig. 7).

### Restricted cubic spline analysis

**Figure 8:**
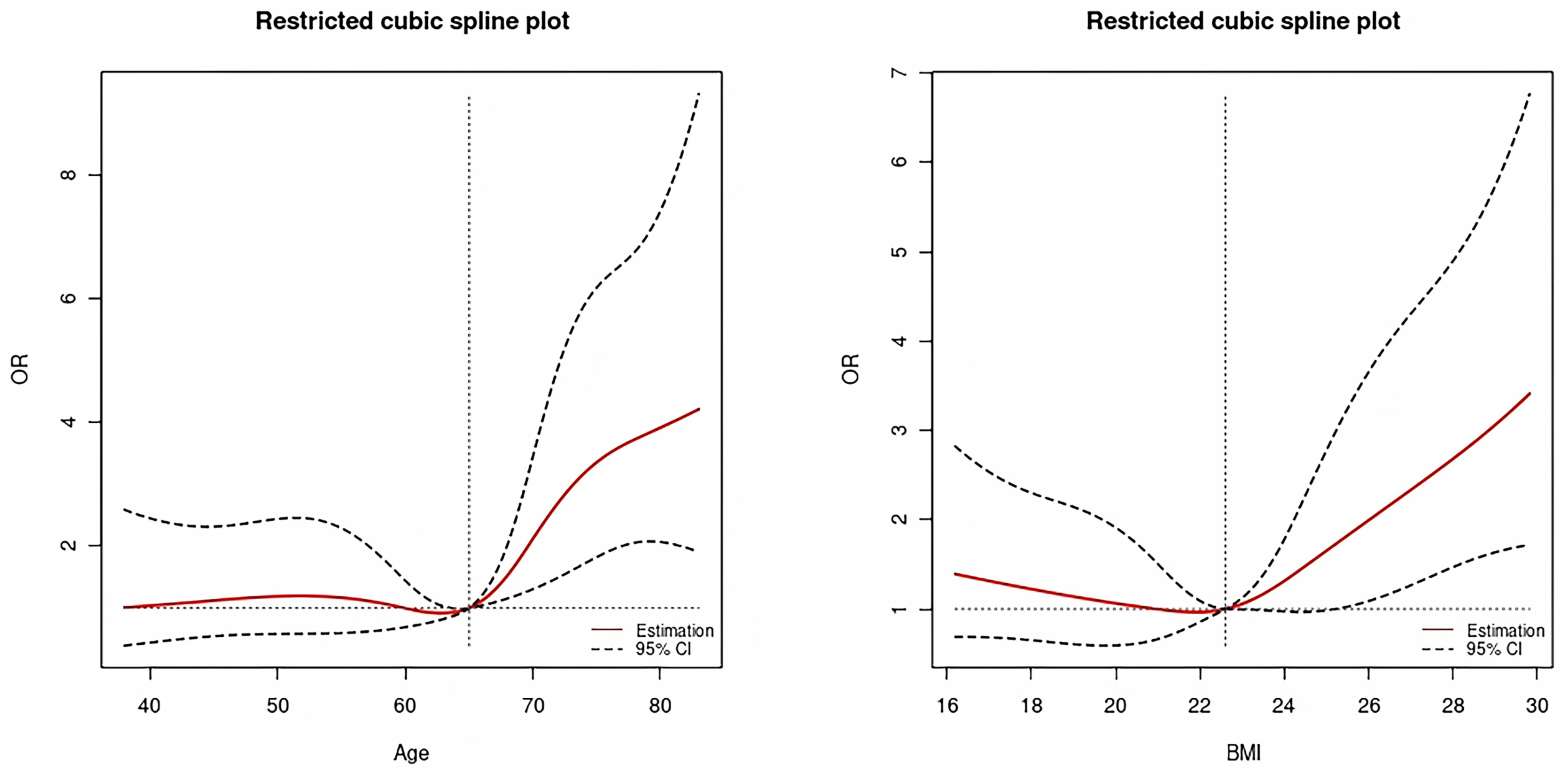
Restricted cubic spline plot of age (left) and BMI (right). The non-linear associations between age (left) and BMI (right) with the odds ratio (OR) of the outcome. The solid red line represents the estimated OR, and dashed lines indicate the 95% confidence interval. Vertical dotted lines mark reference values used for comparison.

Among the independent risk factors identified in this study, age and BMI were continuous variables. To further elucidate their potential nonlinear associations with the risk of postoperative pleural effusion, restricted cubic spline (RCS) modeling was applied for visual representation (Wang et al., 2025b). The RCS analysis revealed distinct nonlinear associations between age, body mass index (BMI), and the risk of postoperative pleural effusion (Fig. 8). Specifically, the incidence of pleural effusion remained relatively stable among patients younger than 65 years. However, beyond 65 years of age, the odds ratio (OR) for pleural effusion increased markedly with advancing age. Regarding BMI, the relationship displayed a U-shaped curve. When BMI was below 22.6, the OR for pleural effusion declined as BMI increased. Conversely, at BMI values above 22.6, the OR rose sharply in parallel with increasing BMI.

## Discussion

Pancreatic surgery remains a key therapeutic approach for managing pancreatic disorders. However, the procedure is associated with a relatively high incidence of postoperative complications, with pleural effusion being among the most frequently encountered (Bustamante Bernal et al., 2017). Pleural effusion not only exacerbates patient discomfort but can also contribute to respiratory impairment and prolonged hospitalization. Consequently, developing predictive tools for pleural effusion and enabling early risk identification may support improved postoperative recovery and enhanced patient well-being.

A small volume of postoperative pleural effusion may represent a reactive process, often occurring without notable clinical symptoms. To ensure diagnostic consistency and focus on clinically relevant cases, this study defined pleural effusion as the presence of effusion accompanied by hypoxemia. This definition highlights a subset of patients with symptomatic pleural effusion, who offer greater value for research targeting postoperative complications and prognostic assessment (Aujayeb, 2022).

Five independent risk factors for postoperative pleural effusion were identified in this study: age, BMI, atrial fibrillation, ASA grade and intraoperative blood transfusion.

Age is a significant risk factor for postoperative pleural effusion. Numerous studies have demonstrated that elderly patients, particularly those aged 70 years and above, are more susceptible to developing pleural effusion following surgery (Hao et al., 2025). In a study of patients undergoing lobectomy, age over 70 years was identified as a significant risk factor for substantial pleural effusion (>400 mL/day) on postoperative day 2 (Hristova et al., 2015). In addition, in patients undergoing cardiac surgery, age has also been confirmed to be related to the occurrence of postoperative pleural effusion (Schiefenhövel et al., 2022). In this study, age was significantly associated with the development of pleural effusion following pancreatic surgery, consistent with findings from previous research. Age-related declines in pulmonary and cardiovascular function may impair postoperative recovery and contribute to this complication. For instance, increased permeability of the alveolar–capillary membrane in elderly individuals facilitates fluid exudation. Moreover, the higher prevalence of chronic conditions such as COPD and heart failure further elevates the risk of postoperative pleural effusion in this population (Feng et al., 2024). RCS analysis in this study revealed a sharp increase in pleural effusion risk among patients over 65 years of age, consistent with findings reported in previous studies.

In this study, the influence of BMI on postoperative pleural effusion exhibited a nonlinear pattern. RCS analysis revealed a U-shaped relationship, indicating elevated risk at both lower and higher BMI ranges. When BMI was below 22.6, the OR for postoperative pleural effusion decreased with increasing BMI. However, once BMI exceeded 22.6, the OR rose sharply as BMI increased further. In a study on esophageal cancer surgery, patients with a BMI below 18.5 exhibited a higher incidence of postoperative pleural effusion compared to those within the normal BMI range. Additionally, the risk of pleural effusion was elevated in obese patients (BMI > 24), suggesting a U-shaped relationship between BMI and postoperative respiratory complications (Ri, Aikou & Seto, 2018). Patients with low body weight often present with poor nutritional status, which may compromise energy availability and nutritional support during the postoperative recovery phase. Such deficiencies can impair wound healing and tissue repair, thereby elevating the risk of pleural effusion. In obese patients, excessive adipose tissue can hinder surgical field exposure and prolong operative time, thereby increasing the likelihood of postoperative complications, including pleural effusion. Additionally, obesity is frequently associated with respiratory dysfunctions, such as obesity hypoventilation syndrome, which may further contribute to impaired pulmonary recovery. These patients are more prone to developing postoperative respiratory insufficiency, which can impair pulmonary ventilation and gas exchange. Such dysfunction may subsequently contribute to the formation of pleural effusion (Anderson & Shashaty, 2021). Excess adipose tissue can compress intrathoracic lymphatic vessels. This compression may impair lymphatic drainage and promote fluid buildup in the thoracic cavity, leading to pleural effusion (Azhar et al., 2020). Both ends of the BMI spectrum may reflect underlying systemic vulnerabilities that predispose patients to postoperative complications. Low BMI may be associated with sarcopenia and impaired immune responses, while high BMI often coexists with metabolic syndrome and systemic inflammation. These factors can exacerbate postoperative stress responses and compromise cardiopulmonary function, thereby reinforcing the observed U-shaped association between BMI and pleural effusion risk.

Atrial fibrillation may act as an independent risk factor for pleural effusion by disrupting cardiovascular hemodynamics and triggering inflammatory cascades. The loss of coordinated atrial contraction reduces cardiac output, potentially leading to left or right heart failure. This dysfunction elevates venous pressure within the pulmonary or systemic circulation, thereby promoting transudative fluid accumulation in the pleural cavity (Li, Chen & Hu, 2022). Atrial fibrillation is commonly associated with comorbid conditions such as pulmonary infections, embolic events, and anticoagulant therapy. These factors may individually or collectively promote the formation of exudative or hemorrhagic pleural effusions (Pokorney et al., 2022; Boos, 2020). Overall, atrial fibrillation facilitates the development of pleural effusion through a range of interrelated pathophysiological mechanisms.

In this study, patients with higher ASA grades were more prone to developing pleural effusions. Multiple studies have demonstrated a significant association between elevated ASA classification and increased risk of postoperative pleural effusion (Meyer et al., 2021). For instance, one study demonstrated that patients with an ASA grade exceeding II faced a significantly elevated risk of postoperative pulmonary complications (Smetana, 2009). Another study has identified ASA grade III as an independent risk factor for postoperative pulmonary complications including pleural effusion following thoracoscopic pneumonectomy (Feng et al., 2024). ASA classification reflects the severity of patients’ preoperative condition. Since pleural effusion is closely associated with overall physiological status, an elevated ASA grade may serves as an independent risk factor for its postoperative occurrence.

In this study, intraoperative blood transfusion emerged as an independent risk factor for postoperative pleural effusion. One possible mechanism is transfusion-related lung injury, where donor antibodies react with the recipient’s white blood cells. This immune activation triggers an inflammatory cascade that damages the pulmonary vascular endothelium, facilitating fluid leakage into the alveolar spaces (Sachs et al., 2011). In addition, substances such as reactive oxygen species and transfusion-related bioactive lipids released during blood transfusion may directly injure pulmonary vascular endothelial cells. These agents can disrupt endothelial integrity, increase vascular permeability, and contribute to fluid extravasation into the pleural space (Rabie et al., 2024). Secondly, intraoperative blood transfusion often indicates significant surgical bleeding. Excessive hemorrhage may impair hepatic perfusion and induce hepatocellular hypoxia. Moreover, transfusion itself may provoke ischemia-reperfusion injury to liver cells, thereby hindering postoperative hepatic recovery. The resulting disturbances in protein synthesis can lead to hypoproteinemia, which facilitates the formation of substantial ascites. Under elevated intra-abdominal pressure, this ascitic fluid may migrate into the thoracic cavity, contributing to pleural effusion (Nastos et al., 2014). Intraoperative blood transfusion often indicates a more complex surgical procedure. Pancreatic surgery, as an upper abdominal intervention, entails prolonged operative time, which may intensify diaphragmatic stimulation and disrupt thoracic integrity. This increased procedural disturbance can contribute to the development of postoperative pleural effusion.

To date, relatively few studies have investigated predictive models for pleural effusion following pancreatic surgery. A prior study (Wang et al., 2025a) conducted by our research group on pulmonary complications after abdominal surgery identified several independent risk factors for postoperative pulmonary complications in critically ill patients. These included advanced age, elevated BMI, sepsis, hypertension, low preoperative albumin levels, increased postoperative lactic acid concentrations, upper abdominal surgical approach, ASA classification ≥III, intraoperative administration of vasoactive agents, and the use of nasogastric tubes. That study enrolled a larger sample size of over 3,000 cases and examined postoperative pulmonary complications (PPCs) as a heterogeneous group of conditions. Consequently, a broader array of risk factors was ultimately identified. In contrast, the present analysis specifically focused on pancreatic surgery, which is among the most technically demanding abdominal procedures. Despite differences in surgical scope, shared risk factors such as advanced age, elevated BMI, and higher ASA classification were consistent across both studies. However, intraoperative blood transfusion and atrial fibrillation emerged as distinct risk factors in the present cohort. These differences may be attributed to the increased likelihood of transfusion during pancreatic surgery and the critically ill status of the patients. In addition, more pronounced hemodynamic fluctuations and a heightened susceptibility to atrial arrhythmias may further contribute to these variations.

Zhou et al. (2022) conducted a study on pleural effusion following severe acute pancreatitis (SAP), enrolling a total of 222 patients. The incidence of pleural effusion in this cohort was 29.28%, which is slightly higher than the 27.8% reported in the present study. This discrepancy may be attributed to the inclusion of exclusively SAP patients, who typically exhibit prolonged hospital stays and intensified inflammatory responses. The predictive model included serum albumin, fibrinogen, C-reactive protein, APACHE II, and SOFA scores—all positively associated with inflammation and disease severity in SAP. In contrast to that study, the majority of patients in our cohort underwent elective surgical procedures, resulting in a markedly different patient profile. Due to data limitations, scores such as APACHE-II and SOFA were not available. Nonetheless, key variables from these scoring systems—reflecting baseline health and lab parameters—were mostly captured through individual data points, reducing the impact of their omission. Furthermore, inclusion of both scores may introduce multicollinearity issues given their overlap with routinely assessed clinical variables. Notably, our study employed RCS analysis to examine the continuous variables of age and BMI, which revealed nonlinear associations with postoperative pleural effusion.

As this study is observational in nature, residual confounding cannot be fully excluded despite statistical adjustment. The associations identified do not imply causality, and unmeasured or unknown confounders may have influenced the observed outcomes. This constraint should be considered when interpreting the predictive model and its clinical implications.

This study has several limitations. First, its retrospective design limited the availability and completeness of clinical data. Second, the relatively small sample size may restrict the generalizability of the findings. Third, the model was internally validated but lacked external validation, which limits its applicability across different clinical settings. And, we acknowledge that our definition about pleural effusion(pleural effusion with hypoxemia) may be narrower than conventional classifications of pleural effusion, but it was intentionally selected to emphasize cases with direct clinical impact. Future large-scale prospective studies with external validation cohorts are needed to confirm these results and strengthen the evidence base. In addition, this study relied on chest X-ray for the diagnosis of postoperative pleural effusion due to the retrospective nature of the dataset, which did not include ultrasound records. While chest X-ray is routinely used in ICU settings, its diagnostic sensitivity is known to be inferior to that of bedside ultrasound. We acknowledge this limitation and recognize that future prospective studies should incorporate ultrasound-based assessments to improve diagnostic accuracy and enhance model reliability.

## Conclusions

In conclusion, we developed a predictive model to assess the risk of pleural effusion following pancreatic surgery. Key predictors included patient age, BMI, atrial fibrillation, ASA grade, and intraoperative blood transfusion. The model demonstrated good discriminatory performance and calibration, supporting its potential utility for early risk stratification and personalized clinical decision-making. Nonetheless, external validation and large-scale prospective studies are needed to confirm its generalizability and assess its broader clinical impact.

## Supplemental Information

10.7717/peerj.20635/supp-1 Supplemental Information 1 STROBE checklist.

10.7717/peerj.20635/supp-2 Supplemental Information 2 Codebook.

10.7717/peerj.20635/supp-3 Supplemental Information 3 Raw data.

## Additional Information and Declarations

### Data Availability

The following information was supplied regarding data availability:

The raw measurements are available in the Supplemental File.
