# Supplementary material for: Retrospective study of postoperative pleural effusion with hypoxemia in critically ill pancreatic surgery patients: model development and restricted cubic spline analysis
Source: PeerJ. 2026 Jan 30;14:e20635. doi: 10.7717/peerj.20635 (PMC12863154; doi:10.7717/peerj.20635)
Supplement: Supplemental Information 2 [file peerj-14-20635-s002.docx]

Age: 1=male, 0=femle

Cardiacinsufficiency: 1=yes, 0=no

Fibrillation: 1=yes, 0=no

Diabetes: 1=yes, 0=no

Hypertension: 1=yes, 0=no

CAD（**coronary heart disease**）: 1=yes, 0=no

Cerebralinfarction: 1=yes, 0=no

Chemotherapy: 1=yes, 0=no

HBV（hepatic B virus）: 1=yes, 0=no

Fattyliver: 1=yes, 0=no

Smoker: 1=yes, 0=no

Laparotomy: 1=yes, 0=no

Multisitesurgery: 1=yes, 0=no

Intraoperativechemotherapy: 1=yes, 0=no

Transfusion: 1=yes, 0=no

Analgisapump: 1=yes, 0=no

Norepinephrine: 1=yes, 0=no

Pleuraleffusion: 1=yes, 0=no

Gender: 1=male, 0=female

COPD: 1=yes, 0=no
